# Proceedings of the Terrell Medical Society

**Published:** 1889-01

**Authors:** E. E. Smiley

**Affiliations:** Secretary and Treasurer


### SOCIETY NOTES.

### PROCEEDINGS OF THE TERRELL MEDICAL SOCIETY.

Dr. John Inabnit in the chair.

Dr. Garret reported the case of a patient suffering from acute articular rheumatism, used the usual vaunted remedies, and to no purpose, finally concluded to add the effect of a electric battery, and in a few days noticed very beneficial effects, and has since concluded that electricity is an excellent adjunct to medical therapeutics in the successful treatment of rheumatism.

Dr. Fowler, as regular contributor read a paper upon the treatment of pneumonia, with veratrum viridi, and also explained his views upon its adaptability in puerperal eclampsia as an inhibitor excitor affecting the *vasso-motor* nerve centres; usually gave the drug till emesis was induced, then following with alcoholic stimulants; results were in every case good, given hypodermically in doses of from five to seven drops hourly, till nausea, or preferably, vomiting ensued.

Dr. Stroud thought nausea and vomiting to be the effect of the drug resulting favorably in all such cases, and held that its effects were not realized until this nausea and vomiting were established.

Dr. Dumas reported a case of cystitis complicated with gastritis; patient age 74, suffered from insomnia, pains in the stomach and bladder, urine coffee color, loss of appetite, and violent hiccoughs, mental faculties intact; used warm water injections, nitrate silver lotions, followed by saline lotions per urethram; obstinate constipation, gave laxatives, relieved condition of bowels, but the hiccough proved to be very obstinate; gave nitrite amyl, then followed its use with ⅛ to ⅙ gr extract hyosciamos, greatly to the benefit and comfort of the patient.

Dr. Nelson reported a case of spinal disease, which he regarded as incurable, but thought such cases should be reported; and their treatment to be instituted early and pushed vigorously, would sometimes result in perfect cures. He cited a case of membranous dysmenorrhœa, in which he removed a large quantity of clots and debris from the vagina, left it properly tamponed, after removing the tampon found quantities of exfoliated membrane. Then using the curette vigorously no more trouble was experienced with the case; recovery good.

Dr. Orr asked if the uterus contracted well.

Dr. Nelson said that it did.

Dr. Fry, of Elmo, reported that the patient, a negro woman, from whose breast, Dr. Nelson Dr. Dumas and he removed a fibroid tumor weighing some eight pounds, was now troubled with a similar tnmor in the other mammary gland, and said that considering their best endeavors to remove all affected glands, he was a little disappointed, as he had told the patient that in all probability there would be no return.

Dr. Smiley read an excellent paper upon the beneficial effects of electricity, and the best manner in which to apply it.

Dr. Nelson cited several cases in which he received excellent results from the use of electricity.

Dr. White thought it an excellent medium through which to treat the mind, as we are frequently called upon to treat mind diseases.

Dr. Fry cited a very obstinate case of obstruction which yielded speedily to the effects of electricity.

Dr. Strain read an able paper upon the use and abuse of the obstetrical forceps; holding that many cases were lost in the parturient act, not because the forceps were used, but because they were not used intelligently.

Dr. Inabnit was not inclined to favor the forceps; thought nature was equal to any and all emergencies; he favored the relaxation of the system and leaving the case in charge of dame nature.

Dr. Smith reported an interesting case of malignant sarcoma. with disease of the nasal passages. Patient had copious hemorrhage from the same, and with much labor he controled it. Upon examination found a tumor so large as to be unable to pass the snare over it, and from the anemic condition, fearing that the hemorrhage and subsequent shock would prevent a proper reaction, had referred the case to the surgical staff of the St. Louis Medical College, who advised the patient to return home and not undergo an operation, as it would prove fatal.

There was quite a sensation created at our last meeting, concerning alleged unprofessional conduct. Such charges are antagonistic to harmony and should never occur. The code is certainly plain enough, though a man be a fool, he need not err therein. No, it is not due to its misinterpretation, but is due to a contemptuous morbid desire to override the just claims of another professional brother whom circumstances seem to favor. The dispositlon to prevent the success of a colleague is too frequently made apparent even to the disgust of a community. It is the supreme object, and it should be the duty of every medical society to expound the principles of etiquette by which physicians are to be governed. Many there are who are continually prating about the code and who know nothing concerning its teachings.

Dr. A. J. Stovall, whose name was dropped some two years ago from the list of members for circulating circulars and advertisments, vaunting a certain cureall, termed Dr. Stovall's Favorite Prescription, has (upon a written application for reinstatement into its membership, renouncing his past conduct as unprofessional and beneath the dignity of a regular physician, and pledging his support to our ranks according to the code of ethics) been readmitted into full membership.

E. E. Smiley, M. D., Secretary and Treasurer.

